# Cardio‐Invasive Metastatic Squamous Cell Carcinoma of the Lung Masquerading as Acute ST Elevation Myocardial Infarction

**DOI:** 10.1002/ccr3.70481

**Published:** 2025-05-07

**Authors:** Nihar Jena, Shreya Tiwary, Deepak Chandramohan, Sreekant Avula, Prashanth Reddy, Harkesh Arora, Geetha Krishnamoorthy, Kirit Patel, Aiden Abidov

**Affiliations:** ^1^ Department of Cardiology Trinity Health Oakland/Wayne State University Pontiac Michigan USA; ^2^ Department of Family Medicine Sparrow Hospital/Michigan State University Lansing Michigan USA; ^3^ Department of Nephrology University of Alabama at Birmingham Birmingham Alabama USA; ^4^ Department of Diabetes, Endocrinology and Metabolism University of Minnesota Minneapolis Minnesota USA; ^5^ Department of Internal Medicine Yuma Regional Medical Center Yuma Arizona USA; ^6^ Department of Internal Medicine Lovelace Medical Center Albuquerque New Mexico USA; ^7^ Division of Cardiology Wayne State University School of Medicine Detroit Michigan USA; ^8^ Cardiology Section John D Dingell VA Medical Center Detroit Michigan USA

**Keywords:** acute myocardial infarction, cardiac metastasis, coronary artery disease, echocardiogram, squamous cell carcinoma

## Abstract

The incidence of secondary cardiac malignancies is on the rise due to the aging population. A 63‐year‐old male presented with recurrent chest pain and anterior ST elevations on ECG, leading to primary PCI, but continued to experience chest pain and persistent ST elevation. Cardiac imaging revealed a cardio‐invasive lung malignancy.

## Graphical abstract

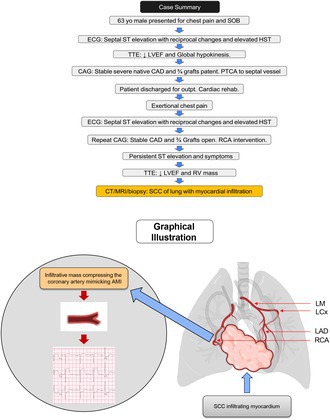

**Summary**

- Cardiac metastasis can cause mechanical compression, which may mimic the symptoms of acute coronary syndrome.
- A comprehensive approach that integrates the patient's medical history, clinical presentation, and various diagnostic methods can help establish an accurate diagnosis and prevent unnecessary procedures.
- Clinicians should encourage patients to undergo recommended screenings for cancer and cardiovascular issues promptly to facilitate early diagnosis.

## 1 Introduction

Myocardial carcinomas are rare, and diagnosis poses significant challenges due to varied clinical presentation. Secondary cardiac malignancies are relatively more frequent than primary ones. Among those, supradiaphragmatic malignancies are the most common to metastasize to the heart. Endocardial involvement is rare, followed by involvement of the pericardium, epicardium, and myocardium. Myocardial metastasis leads to varied clinical presentations ranging from arrhythmias, congestive heart failure, myocardial infarction, and intracavitary mass lesions [1]. External mass compression can lead to myocardial ischemia, leading to unwanted procedures, especially in patients with known coronary artery disease. This article highlights a patient with a prior diagnosis of coronary artery disease (CAD) and Coronary artery bypass graft (CABG) presenting as recurrent ST‐elevation myocardial infarction (STEMI) leading to multiple cardiac interventions but later diagnosed with cardiac metastasis from lung squamous cell carcinoma (SCC).

## 2 History

A 63‐year‐old with a history of CAD with CABG, peripheral arterial disease with multiple interventions, paroxysmal atrial flutter, hypertension, dyslipidemia, diabetes mellitus type 2, deep vein thrombosis, and ischemic stroke with residual aphasia. The patient had a lung cancer screening with low‐dose CT a year ago without suspicion of malignancy. He presented from cardiac rehabilitation (CR) with acute onset chest pain and shortness of breath. The patient underwent CR after an acute myocardial infarction 1 week ago. During that time, he had a similar presentation with chest pain and dyspnea and was diagnosed with anteroseptal STEMI. Emergent percutaneous coronary intervention (PCI) revealed severe stable CAD with patent left internal mammary artery to left anterior descending artery (LAD), saphenous vein grafts (SVG) to obtuse marginal, SVG to the diagonal artery, and occluded SVG to right coronary artery (RCA). Considering the ongoing symptoms, a large septal branch was revascularized. The patient became symptom‐free, and ECG normalized. Following his discharge at CR, he experienced similar symptoms and was sent to the emergency department for further evaluation.

## 3 Investigations

**FIGURE 1:**
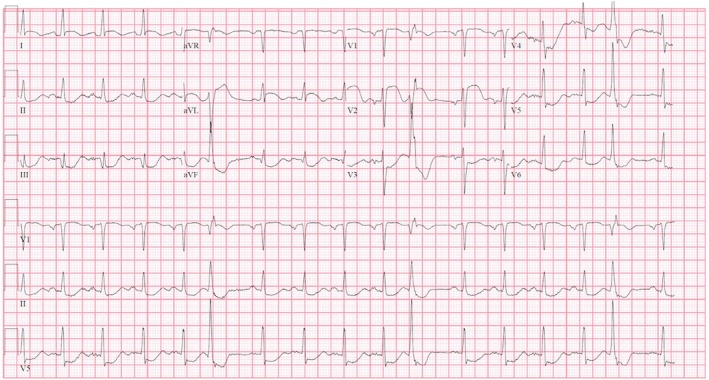
ECG during first presentation showing ST segment elevation in leads I, aVL, V1, and V2. ST depression in leads V3, V4, V5, II, III, and aVF.

**FIGURE 2:**
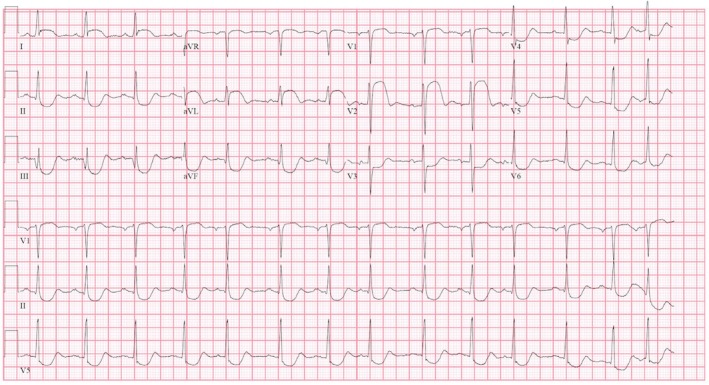
ECG during the second presentation showing ST segment elevation in leads I, aVL, V1, and V2. ST depression in leads V3, V4, V5, II, III, and aVF.

**FIGURE 3:**
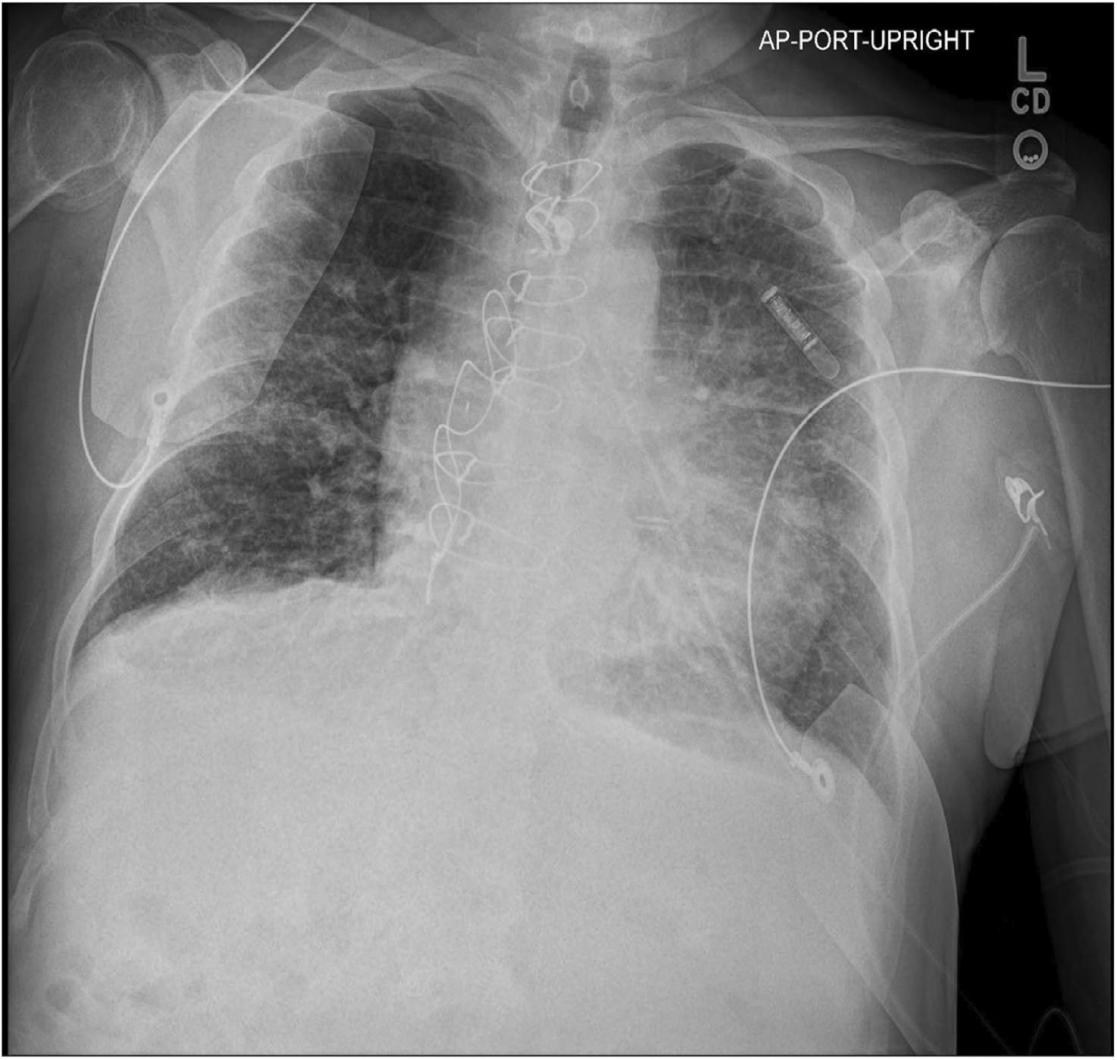
Chest X‐ray showing nonspecific findings of bilateral diffuse opacities and increased bronchovesicular markings.

ECG showed septal ST‐segment elevation with reciprocal depression in the inferior and lateral leads (Figures 1 and 2). His high‐sensitivity troponin was 115 ng/L. Chest x‐ray showed bilateral atypical infiltrate superimposed with pulmonary edema (Figure 3).

## 4 Procedure

**FIGURE 4:**
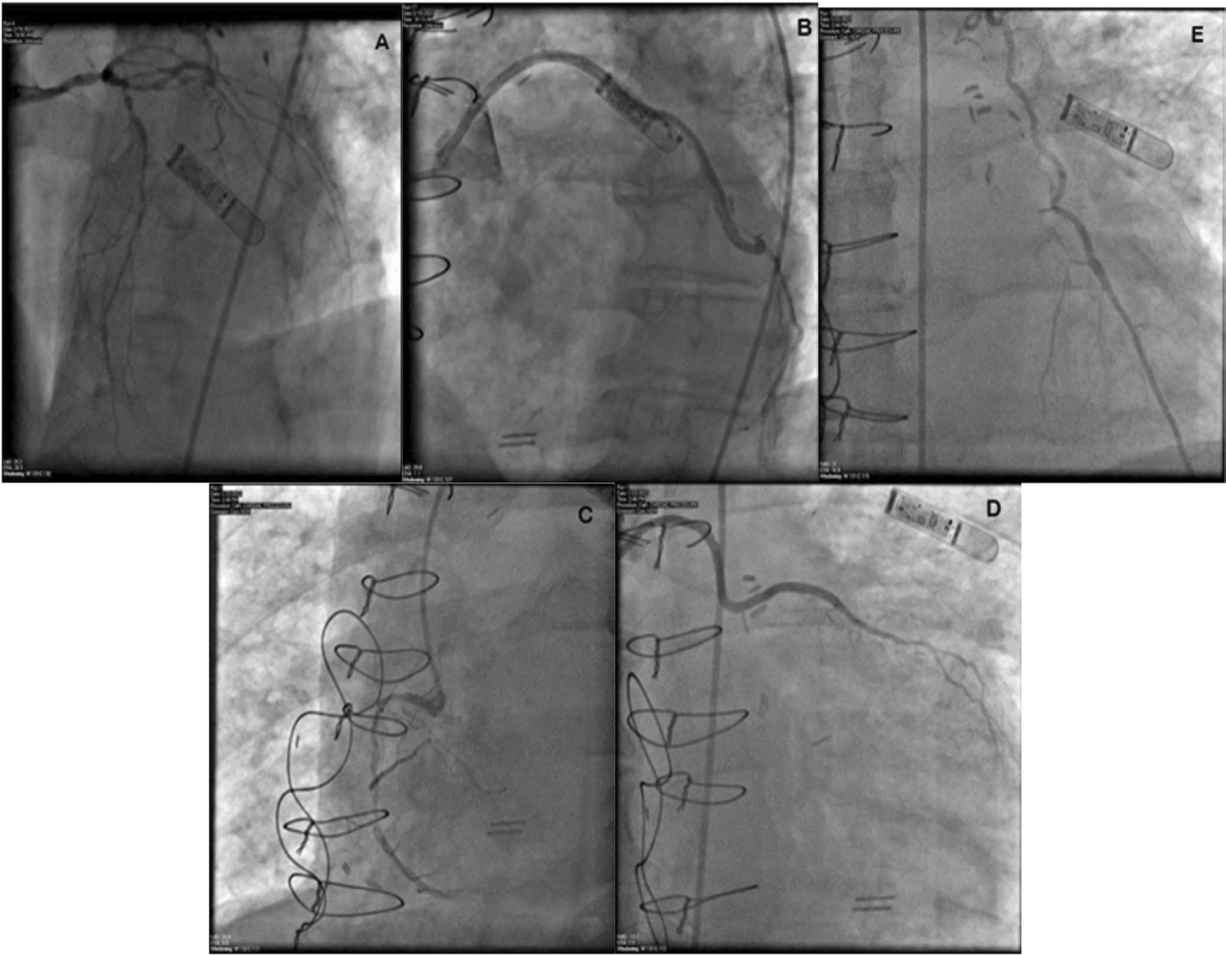
(A–E) Coronary angiogram showing severe native CAD, patent LIMA graft, and SVG grafts.

**FIGURE 5:**
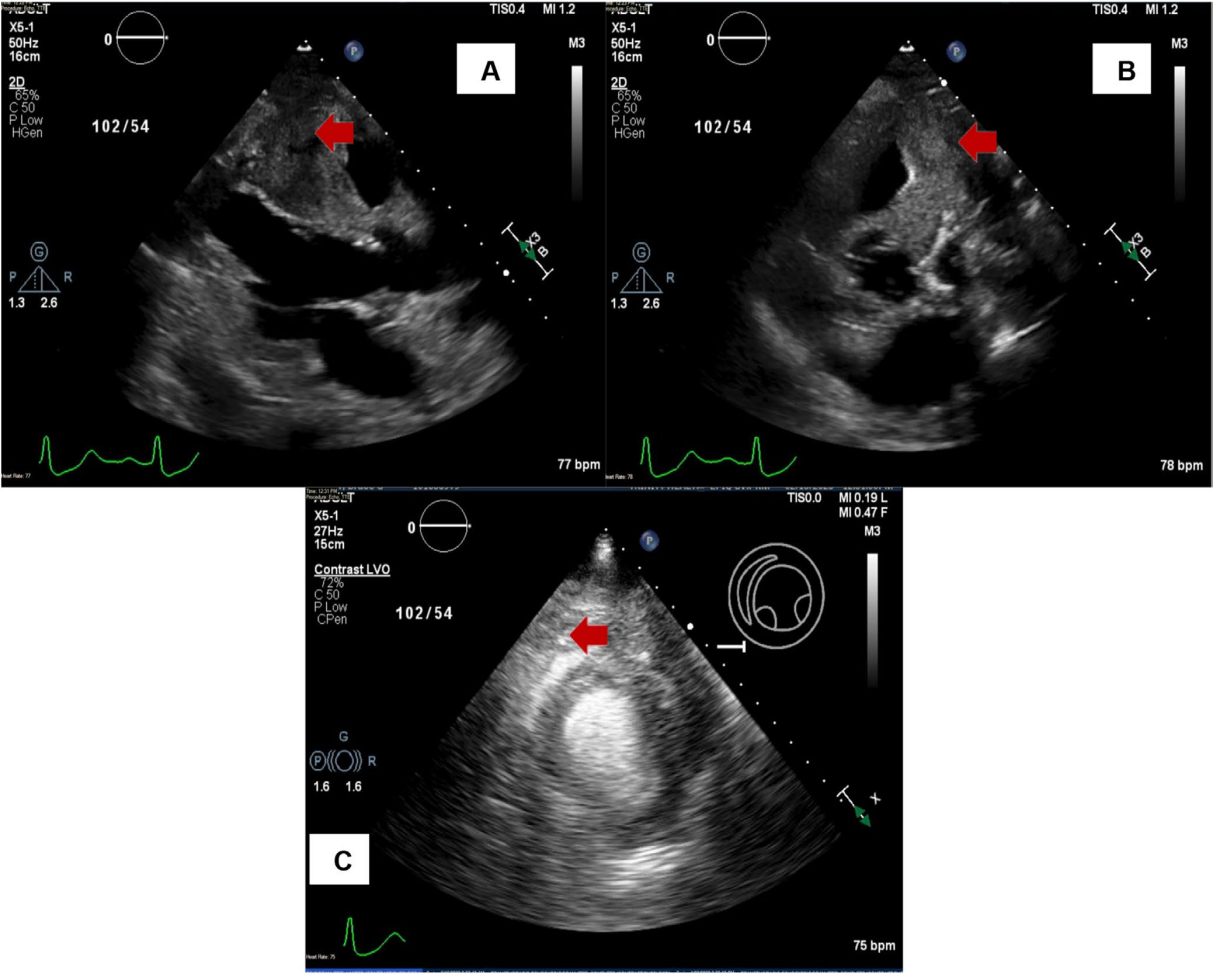
(A–C) TTE‐PSLA/PSSA view showing mass encroachment (red arrow).

**FIGURE 6:**
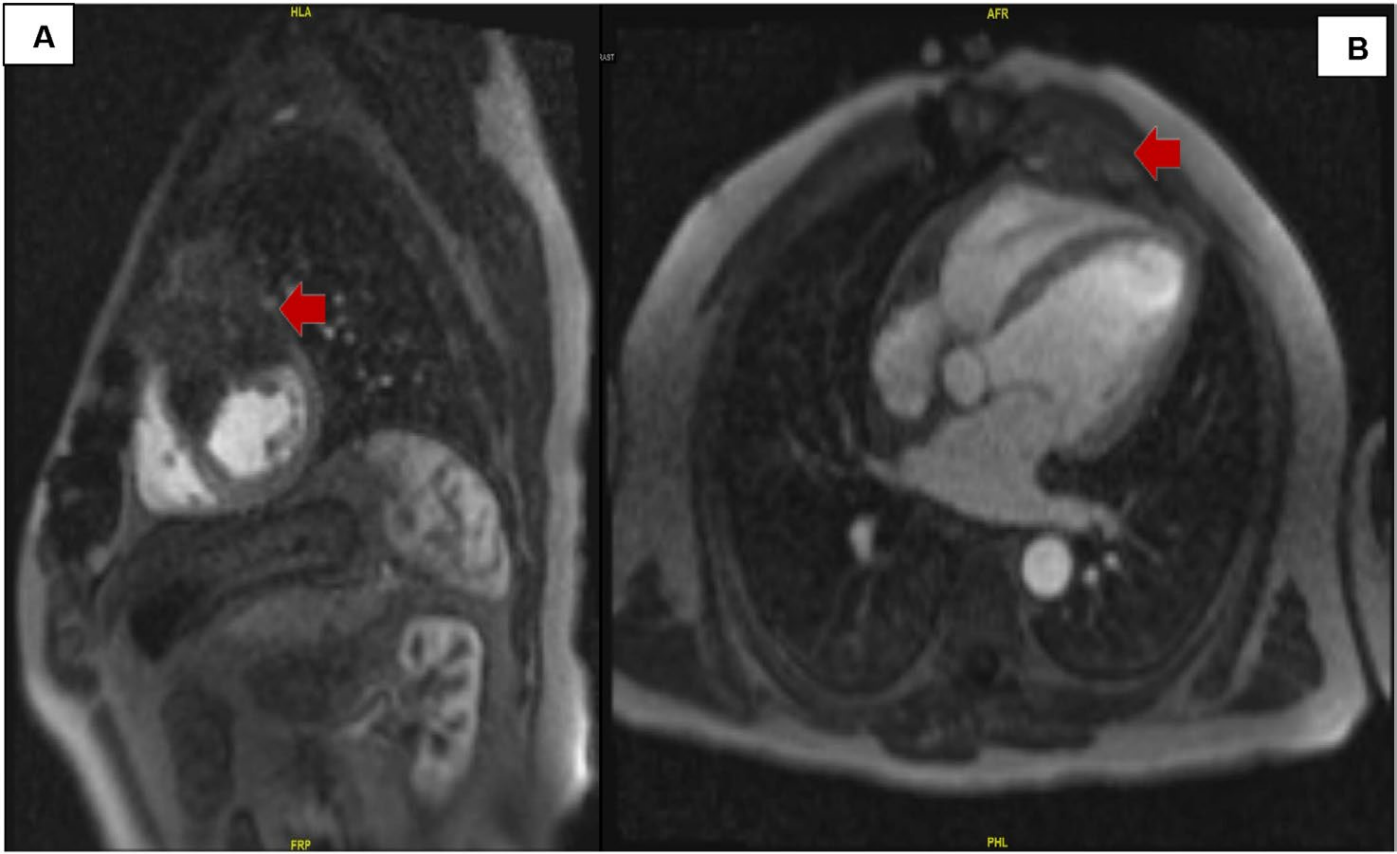
(A, B) Cardiac MRI revealing anterior mass invading the cardiac chambers (red arrow).

**FIGURE 7:**
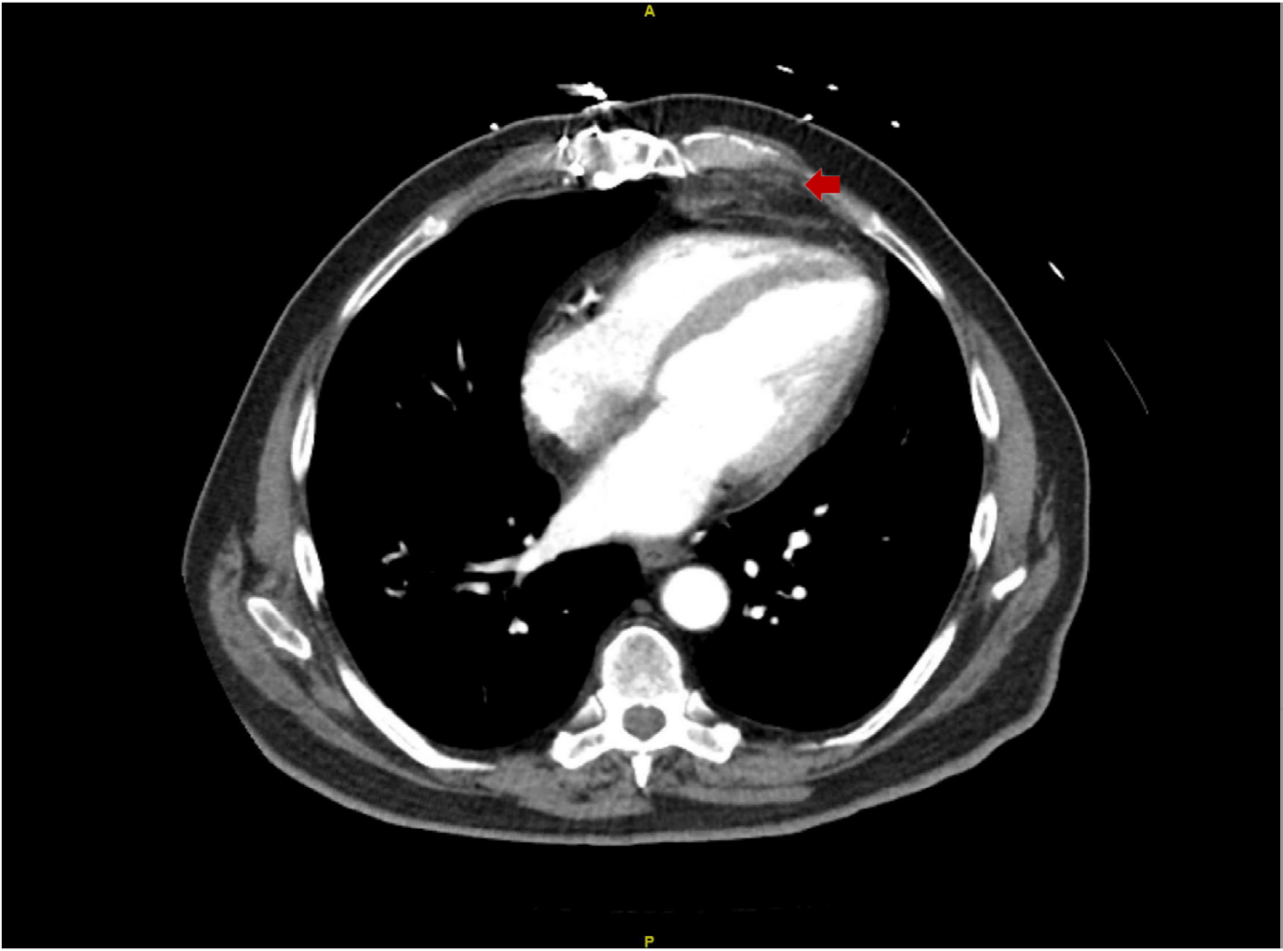
CT of chest showing anterior thoracic mass (red arrow).

**FIGURE 8:**
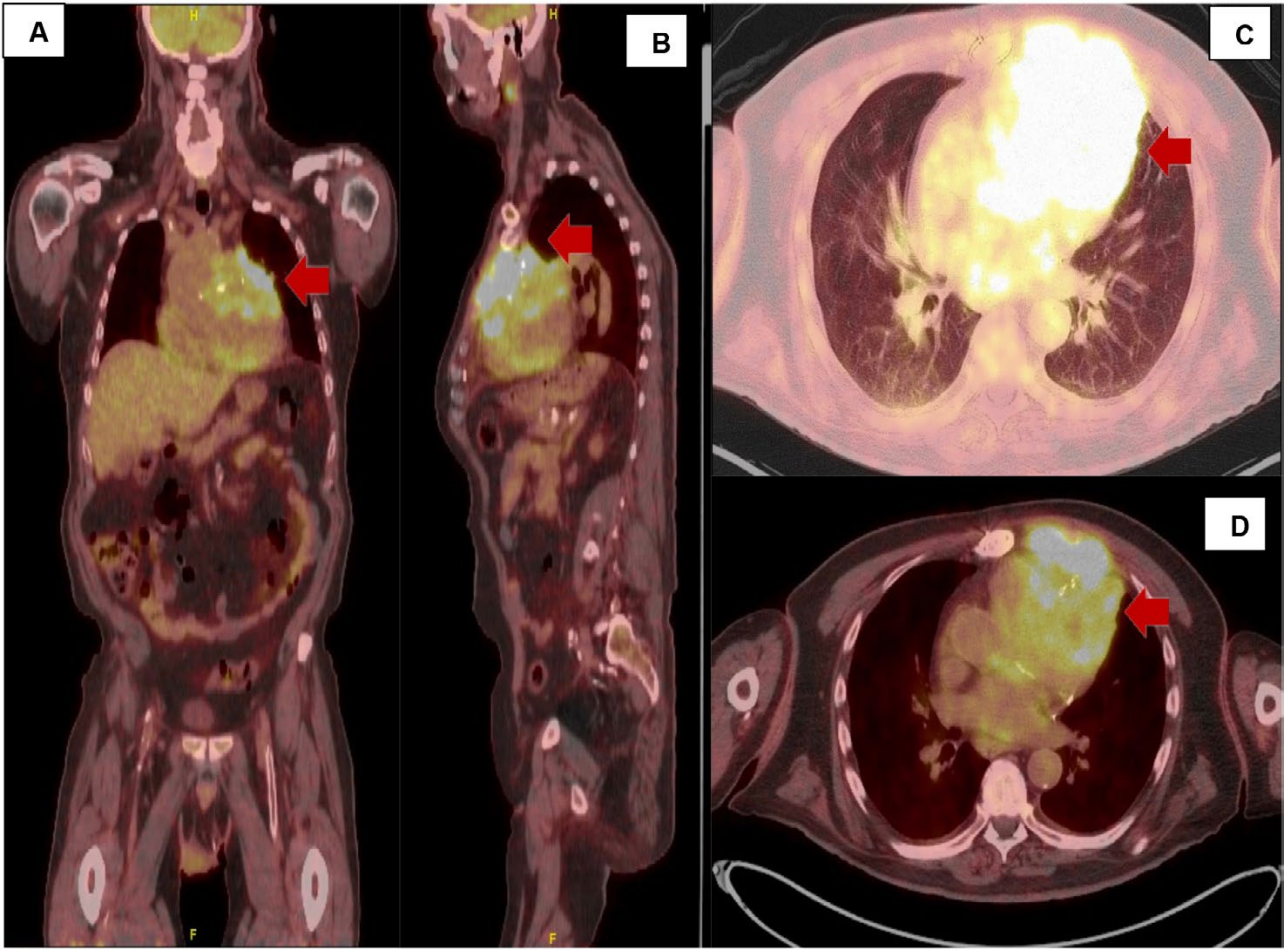
(A–D) PET CT confirms the presence of anterior thoracic mass invading the cardiac structure (red arrow).

The patient underwent emergent cardiac catheterization, showing similar findings as the last angiogram; however, due to persistent chest pain, revascularization of the RCA with two drug‐eluting stents (Figure 4A–E) was done. He had persistent symptoms despite revascularization. A transthoracic echocardiogram (TTE) revealed reduced LVEF at 38% and a mass compressing the right ventricular apex (Figure 5A–C). A cardiac magnetic resonance imaging (MRI) showed a large fungating invading mass on the superior left upper lung field near the bifurcation of the pulmonary artery (Figure 6A,B). This mass had originated outside the pericardium, infiltrating the surrounding structures, including the superior aspect of the right ventricle, pulmonary artery, and mediastinal tissue. A chest computed tomography (CT) demonstrated a large invasive anterior mediastinal mass measuring 10.5 × 8.4 × 6.9 cm, consistent with MRI (Figure 7). CT‐guided biopsy finally revealed malignant SCC. A positron emission tomography scan and brain MRI revealed metastases to the brain and adjacent lymph nodes (Figure 8A–D). Chemotherapy was not tolerated well due to severe axonal neuropathy. Following an extensive discussion about the prognosis, the family and the patient opted for hospice care.

## 5 Discussion

Lung cancer is the most common malignancy worldwide, contributing to 1.8 million deaths in 2018 [2]. This cancer typically metastasizes to the liver and adrenal glands via hematogenous spread [3]. Metastatic cardiac masses usually affect the pericardium due to preferential metastatic pathways involving lymphatics. Myocardial metastatic involvement from lung cancer is infrequent [4]. Histopathologic differentiation of lung malignancy has a differential predilection for cardiac metastases, with lung adenocarcinoma contributing to 21% and lung SCC contributing 18.2% of total secondary cardiac tumors [1]. The most common presentation of patients with cardiac metastasis depends on the type of involvement and the anatomic location. Patients can present with dyspnea, pedal edema, chest pain, palpitation due to various arrhythmias, and life‐threatening presentations such as syncope, stroke, cardiac tamponade, cardiac rupture, acute myocardial infarction, pulmonary embolism, and sudden cardiac death [5]. Abe et al. 1991, conducted a study on 151 lung cancer patients; 11.9% of these patients had myocardial involvement [6]. Similarly, Cate et al., 1986 performed a retrospective analysis on 1046 patients and reported that new onset ECG changes highly suggest myocardial involvement in a clinically stable patient with malignancy [7]. However, it is essential to remember that cardiac involvement is asymptomatic in most patients diagnosed postmortem, precluding obtaining an ECG. TTE remains the diagnostic modality of choice, followed by confirmation with additional imaging and biopsy.

The described patient had significant risk factors for lung cancer, including smoking and age. However, he had a screening CT scan done 1 year ago with normal findings. Chest x‐rays from the current and last admission showed nonspecific conclusions and failed to demonstrate a lung mass. During his first “STEMI,” the patient presented with chest pain and ST‐segment elevation with reciprocal changes and elevated cardiac biomarkers, revealing stable severe CAD with patent grafts. However, after the initial PCI, the patient's condition improved, and other diagnostic studies did not suggest malignancy, so he was discharged. In his second admission with “STEMI,” he had a similar presentation, leading to a coronary angiogram. The RCA was revascularized, assuming it must be the culprit lesion. A subsequent TTE showed suspicion of a cardiac mass, leading to a cardiac MRI and biopsy, revealing the diagnosis of invasive SCC of the lung metastasizing to the myocardium. The following pathways are identified for secondary cardiac metastasis: (a) hematogenous, (b) lymphatic, (c) transvenous, and (d) direct extension. The hematogenous route leads to myocardial involvement. The lymphatic and direct extension lead to pericardial involvement [8]. The described patient has myocardial involvement, indicating hematogenous metastasis. However, the patient had adjacent lymph node involvement but, surprisingly, no pericardial involvement.

The left upper lung mass remained undiagnosed despite multiple prior imaging modalities, including screening low‐dose CT scans and numerous chest X‐rays. A TTE in the presence of recurrent symptoms and persistent ST‐segment elevation led to further investigations and diagnosis. In the described case, due to the presence of severe native CAD, it was difficult to exclude ischemic etiology. Patients with normal coronary arteries or mismatched ST elevation should undergo further investigations before discharge.

Diagnosis of cardiac masses poses a diagnostic challenge, but careful clinical and radiological assessment can aid the diagnosis. Imaging is crucial to detect cardiac masses and metastasis. Echocardiography and MRI are commonly utilized. Delineation and diagnosis of a cardiac mass with echocardiography alone are difficult since the common cardiac masses encountered are usually due to thrombi or vegetation [9]. Myocardial contrast echocardiography, although better, has its limitations. Compared to an echocardiogram, MRI offers better contrast resolution and has the added benefit of identifying intramyocardial masses [10]. Cardiac tumors exhibit low signal intensity on T1‐weighted images and high intensity on T2‐weighted images on MRI, which can differentiate tumors and thrombi [11]. Further assessment, if required, can be performed using the inversion recovery sequence with late gadolinium enhancement [10].

## 6 Conclusion

The case emphasizes the importance of differential diagnosis when patients present with recurrent STEMI‐like symptoms despite optimal invasive treatment. The patient was finally diagnosed within 3 weeks of the first presentation. Cardiac involvement is a late presentation of metastatic lung cancer, so the outcome might not change, but unnecessary invasive studies could have been avoided. Discussing the management of invasive lung carcinoma with cardiac metastasis is beyond the scope of this article.

## Author Contributions

**Nihar Jena:** conceptualization, validation, writing – original draft, writing – review and editing. **Shreya Tiwary:** resources, writing – original draft. **Deepak Chandramohan:** resources, writing – original draft, writing – review and editing. **Sreekant Avula:** resources, writing – original draft, writing – review and editing. **Prashanth Reddy:** resources, validation, writing – original draft. **Harkesh Arora:** resources, writing – original draft. **Geetha Krishnamoorthy:** resources, supervision, validation. **Kirit Patel:** resources, supervision, validation. **Aiden Abidov:** resources, supervision, validation, visualization.

## Ethics Statement

The authors have nothing to report.

## Consent

Written informed consent was obtained from the patient to publish this report in accordance with the journal's patient consent policy.

## Conflicts of Interest

The authors declare no conflicts of interest.

## Data Availability

The authors have nothing to report.
